# Carbon dots conjugated nanocomposite for the enhanced electrochemical performance of supercapacitor electrodes†

**DOI:** 10.1039/d1ra08045h

**Published:** 2021-12-13

**Authors:** Sally M. Youssry, M. Abd Elkodous, Go Kawamura, Atsunori Matsuda

**Affiliations:** Department of Chemistry, Faculty of Science, Tanta University Tanta 31527 Egypt; Department of Electrical and Electronic Information Engineering, Toyohashi University of Technology 1-1 Hibarigaoka, Tempaku-cho Toyohashi Aichi 441-8580 Japan matsuda.atsunori.hh@tut.jp

## Abstract

Naturally, a combination of metal oxides and carbon materials enhances the electrochemical performance of supercapacitor (SC) electrodes. We report on two different materials with highly conductive carbon dots (CDs) and a Co_0.5_Ni_0.5_Fe_2_O_4_/SiO_2_/TiO_2_ nanocomposite with a high power density, a high specific surface area, and a nanoporous structure to improve power and energy density in energy storage devices. A simple and low-cost process for synthesizing the hybrid SC electrode material Co_0.5_Ni_0.5_Fe_2_O_4_/SiO_2_/TiO_2_/CDs, known as CDs-nanocomposite, was performed *via* a layer-by-layer method; then, the CDs-nanocomposite was loaded on a nickel foam substrate for SC electrochemical measurements. A comparative study of the surface and morphology of CDs, the Co_0.5_Ni_0.5_Fe_2_O_4_/SiO_2_/TiO_2_ nanocomposite and CDs-nanocomposite was carried out using X-ray diffraction (XRD), scanning electron microscopy (SEM), transmission electron microscopy (TEM), energy-dispersive X-ray spectroscopy (EDX), BET surface area, and Raman spectroscopy. The synthesized CDs-nanocomposite electrode material displayed enhanced electrochemical performance, having a high specific capacitance of 913.7 F g^−1^ at a scan rate of 5 mV s^−1^ and capacitance retention of 72.2%, as well as remarkable long-life cyclic stability over 3000 cycles in the three-electrode setup and 1 M KOH electrolyte. It also demonstrated a superior energy density of 130.7 W h kg^−1^. The improved electrochemical behavior of the CDs-nanocomposite for SC electrodes, together with its fast and simple synthesis method, provides a suitable point of reference. Other kinds of metal oxide nanocomposites can be synthesized for use in energy storage devices.

## Abstract 2

Schematic diagram for the detailed steps of loading CDs-nanocomposite on nickel foam (NF) substrate to enhance the electrochemical performance of supercapacitor (SC) electrodes.

## Graphical abstract

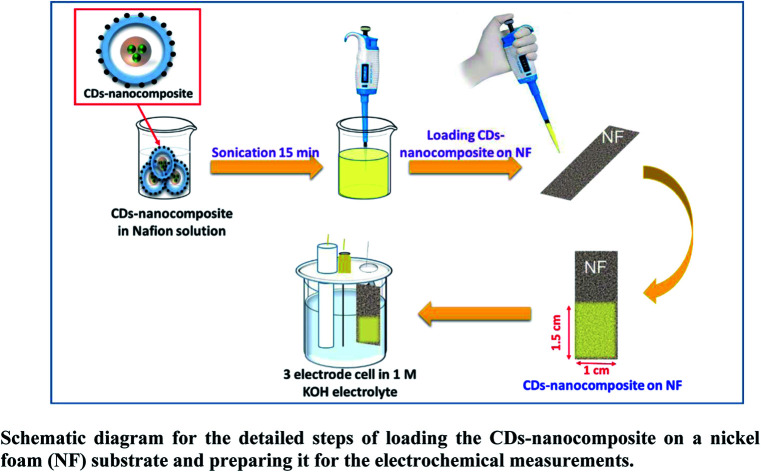

## 1. Introduction

Over the last century, the energy used by humans has come primarily from fossil fuels. Conventional fossil fuel resources continue to be consumed at an alarming rate to meet soaring energy demands. Global climate change caused by the excessive burning of fossil fuels has worsened and become an urgent problem.^1–3^ Consequently, researchers seek new forms of renewable energy to reduce carbon dioxide emissions and enable the transition to clean and eco-friendly energy sources.^4,5^ Such technologies also help to control and reduce the cost of electricity, improve the consistency and flexibility of electrical systems, replace aging power infrastructure, and provide consistent power to remote areas.^6^ However, renewable energy sources are prone to fluctuations. Therefore, energy storage systems will be required to buffer these fluctuations to ensure a stable energy supply.^7–9^

Supercapacitors (SCs), unlike batteries or fuel cells, are the key to efficient electrochemical energy storage as they demonstrate high power density, a fast charge–discharge rate, a long life cycle, and a simple operational mechanism.^10,11^ These properties come from two common mechanisms: the electric double-layer capacitance (EDLC) and the pseudocapacitance (PC). In EDLC, the capacitance comes from the high charge accumulation rate at the electrolyte–electrode interface. Therefore, a large surface area and porous structure are required in this case. In contrast, the PC mechanism employs a fast oxidation–reduction reaction.^12–14^

Activated carbon-based materials, such as carbon nanotube (CNT), carbon dots (CDs), and graphene, are commonly used in the construction of EDLC electrodes due to their high porosity, low cost, chemical and physical stability, and ease of production from renewable sources.^15–17^

However, the specific capacitance of activated carbon electrodes is still lower than that of many other materials, such as conductive polymers and inorganic oxides, which are generally used in PC electrodes.^18^ Therefore, the electrical performance of carbon-based SCs must improve before they are useful for practical applications. This can be accomplished by incorporating pseudocapacitive properties and combining carbon-based materials with materials that have a large surface area and appropriate pore size, such as metal oxides, metal hydroxides, and polymers, or with materials containing oxygen or nitrogen functional groups. Such energy storage systems are known as hybrid SCs.^19,20^

Titanium dioxide (TiO_2_) is widely used as a photocatalyst and semiconductor in dye-sensitized solar cells. However, researchers recently turned their attention to using TiO_2_ as an electroactive material for SCs. TiO_2_ possesses semiconducting properties, low-cost production, high stability, and is environmentally friendly. However, TiO_2_ has some drawbacks, such as slow ion diffusion and low electrical conductivity, which worsen its performance. Its low specific capacitance range of 90–120 µF cm^−2^ is much lower than those of other transition metal oxides (*e.g.* Co(OH)_2_, Co_3_O_4_, NiOH_2_, spinel cobaltites *etc.*).^21–26^

TiO_2_ performance must improve to enable the production of low-cost, practical TiO_2_-based SCs. This can be achieved by combining TiO_2_ with a range of carbon-based materials or conjugated polymers to form composites with mutual EDLC and PC properties, enhancing the SC electrode materials.^27^ TiO_2_-based CDs demonstrate excellent suitability for supercapacitor applications due to their nanometer size, unique electronic and surface functionalities, large surface area, simple synthesis, low environmental risk, and high charge–discharge rate.^28,29^ The hybridization of carbon allotropes with metal oxides will improve the hybrid system's conductivity and structural stability. TiO_2_-based CDs has excellent electron mobility, conductivity, high transmittance, Young's modulus, and large SSA. As a result, it has been used in energy storage applications as an additive with TiO_2_ electrodes.^30–32^

In this study, TiO_2_-based CDs nanocomposite (Co_*x*_Ni_1−*x*_Fe_2_O_4_; *x* = 0.5/SiO_2_/TiO_2_/CDs), termed CDs-nanocomposite, is synthesized *via* a layer-by-layer method. Loading CDs-nanocomposite electrode materials on nickel foam (NF) substrate improves supercapacitive performance. The experimental results display superior specific capacitance magnitudes of 913.7 F g^−1^ at a scan rate of 5 mV s^−1^ and capacitance retention of 72.2%. In addition, the results demonstrate a higher energy density of 130.7 W h kg^−1^ and exceptional long-life cyclic stability over 3000 cycles. CDs-nanocomposite electrode materials also demonstrate significant values of power and energy density. Consequently, this makes CDs-nanocomposite on NF a promising candidate for use as a hybrid supercapacitor electrode material.

## 2. Materials and methods

### 2.1. Materials

Ascorbic acid (C_6_H_8_O_6_), titanium(iv) isopropoxide (97%, C_12_H_28_O_4_Ti), copper acetate monohydrate (C_4_H_8_CuO_5_), tetraethyl orthosilicate (TEOS, 98%, Si(OC_2_H_5_)_4_), ammonium hydroxide (28%, NH_4_OH), hydroxypropyl cellulose (MW = 80 000), absolute ethanol (99.9%, C_2_H_5_OH), cobalt chloride (CoCl_2_), nickel chloride (NiCl_2_), sodium hydroxide pellets (NaOH), and ferric chloride hexahydrate (FeCl_3_·6H_2_O), were purchased from Sigma Aldrich (Germany). All reagents were of extra-pure grade and were used as received from the supplier without further purification.

### 2.2. Preparation of the composite matrix

Co_0.5_Ni_0.5_Fe_2_O_4_/SiO_2_/TiO_2_ nanocomposite matrix was prepared using a layer-by-layer method. Co_*x*_Ni_1−*x*_Fe_2_O_4_; *x* = 0.5 core was firstly-prepared using a coprecipitation route. Cobalt chloride (12.5 mg), ferric chloride 45% (0.05 ml), and nickel chloride (12.5 mg) were mixed with deionized water (D.I.W.) (50 ml) at 80 °C. Then, pH was adjusted to 8 using few drops of NaOH aqueous solution (2 M) leading to the precipitation of ferrite black particles. Then, formed particles were washed using D.I.W and dried at 70 °C for 3 h. Finally, dried particles were calcined for 4 h at 300 °C. Secondly, core–shell structure (ferrite/SiO_2_) was prepared. Ferrite powder (180 mg) (obtained in the previous step) was dispersed in (64 ml) D.I.W. *via* water-bath sonication for 45 min. Then, absolute ethanol (320 ml) and ammonia solutions (25%) (8 ml) were added directly into the dispersion at room temperature. After mixing, TEOS (3.2 ml) was added dropwise to the mixture, which was left under stirring for 16 h. The precipitate was collected by centrifugation and was washed with D.I.W. and ethanol. Finally, the precipitate was dried at 50 °C in air. To load TiO_2_ layer onto the formed core–shell structure, (obtained powder from the previous step) was dispersed in a mixture of absolute ethanol (100 ml), hydroxypropyl cellulose (0.2 g), and (0.48 ml) D.I.W. *via* sonication for 30 min. Then, dissolved titanium(iv) isopropoxide (4 ml) in absolute ethanol (18 ml) was dipped directly into the mixture under vigorous stirring at 85 °C. The reaction was left under reflux conditions for 100 min. The obtained powder was collected and washed with ethanol, then it was re-dispersed in D.I.W. (20 ml) to partially etch the silica layer to form a hollow structure. The dispersion was mixed with (3.5 ml) NaOH solution (2 M) under fixed stirring for 1 h. The powder was then collected, washed several times with D.I.W. and dried for 4 h at 90 °C. Finally, dried powder was calcined for 4 h at 550 °C.

### 2.3. Preparation of CDs

CDs were prepared using a hydrothermal method. In brief, ascorbic acid (6.8 g) was dissolved in (400 ml) D.I.W. Then, copper acetate monohydrate (0.8 g) was added to the formed solution at room temperature under fixed stirring for 10 min. Reaction temperature was then increased gradually to 90 °C and the mixture was left for 5 h. The formed supernatant was purified through Millipore filter paper (0.22 µm). Finally, supernatant containing CDs was freeze-dried for 12 h.

### 2.4. Preparation of CDs-nanocomposite

Prepared CDs and composite matrix (1 : 10 wt/wt) were dispersed in super dehydrated ethanol solution (50 ml) using water bath sonication for 30 min. Next, (1 ml) of NH_4_OH 25% solution was added into the mixture which was kept at vigorous stirring overnight. Finally, collected powder was washed many times using D.I.W. and dried for 6 h at 80 °C.

### 2.5. Loading of CDs-nanocomposite electrode materials on NF substrate for hybrid supercapacitor application

**Fig. 1:**
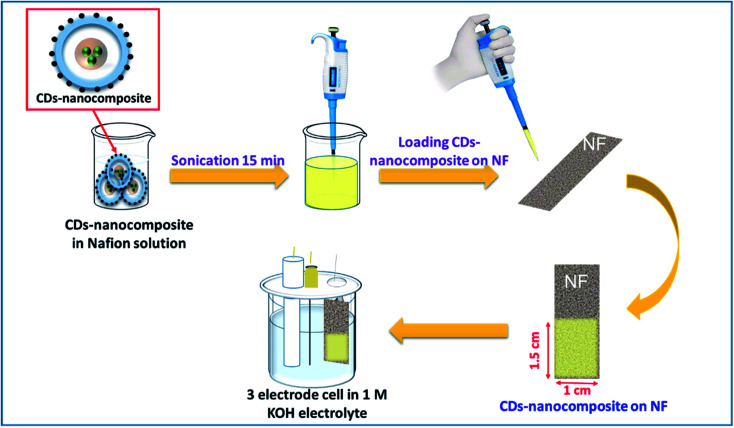
Schematic diagram for the detailed steps of loading CDs-nanocomposite on nickel foam (NF) substrate and preparing it for electrochemical measurements.

Initially, 1 mg of CDs-nanocomposite powder was dispersed in a solution of 1 ml isopropanol, 1 ml deionized water, and 50 µl Nafion (0.5 wt%) and sonicated for 30 min until homogeneous. Then, using a micropipette, 0.5 mg of this solution was loaded on a clean NF substrate of a defined area of 1.5 ×× 1.5 cm^2^. Finally, the NF loaded with CDs-nanocomposite was heated in an oven at 60 °C for 2 h or until completely dry. The CDs-nanocomposite on NF electrode was then ready to be used for electrochemical measurements as shown in the schematic diagram in Fig. 1.

### 2.6. Characterization of the prepared samples

X-ray diffraction (XRD) patterns were recorded using a Rigaku Ultima IV X-ray diffractometer (Japan) with Cu Kα radiation (*λ* = 1.5418 Å) operating at 40 kV and 60 mA. While, SEM analysis was performed *via* SEM (Hitachi SU 8000 Type II) connected with energy-dispersive X-ray spectroscopy (EDX). In addition, BET surface area and BJH pore size distribution analyses were conducted by a Tristar II analyzer (Micromeritics, Japan). In addition, TEM analysis was carried out *via* JEM-2100F (JEOL, Japan). Moreover, Raman analysis was performed on a JASCO NRS-3100 micro-spectrometer with excitation radiation.

### 2.7. Electrochemical analysis

The electrochemical performance of the electrode materials was tested in a three-electrode configuration system using a Solartron SI 1286 electrochemical workstation with 1 M KOH as an electrolyte.

## 3. Results and discussion

### 3.1. Characterizations

#### 3.1.1 XRD analysis

**Fig. 2:**
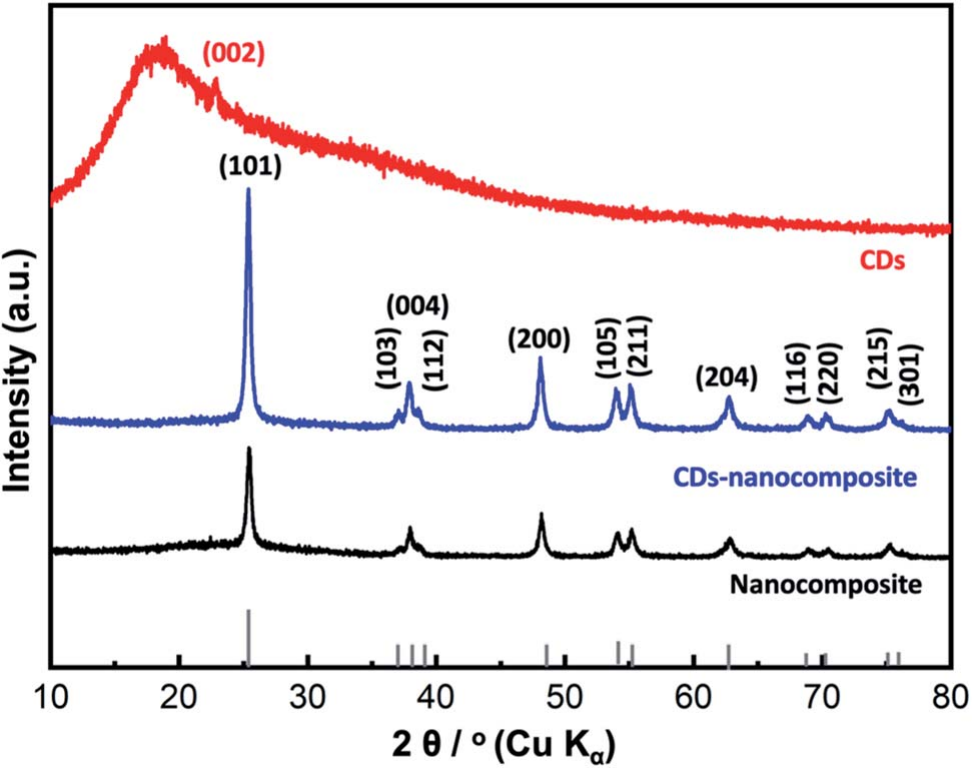
XRD analysis of CDs, nanocomposite, and CDs-nanocomposite.

The formed phase and crystallinity of the synthesized samples were investigated by XRD analysis as shown in Fig. 2. The XRD pattern of the bare composite matrix displayed many diffraction peaks, such as peaks at 2*θ* = 25.6° (101), at 2*θ* = 37.4° (004), at 2*θ* = 48.2° (200), at 2*θ* = 53.6° (105), at 2*θ* = 55.4° (211), at 2*θ* = 62.8° (204), at 2*θ* = 68.8° (116), at 2*θ* = 71.4° (220), and at 2*θ* = 75.4° (215). The observed Bragg peaks and their corresponding crystallographic planes were in good accordance with the anatase TiO_2_ phase (JCPDS 21-1272). Therefore, the TiO_2_ layer is the dominating material in the composite matrix. It is worth noting that the amorphous halo of the SiO_2_ material, usually detected at 2*θ* = 21.5°, was suppressed due to the sharp peaks of the crystalline TiO_2_ layer. Similarly, the corresponding peaks of the Co_0.5_Ni_0.5_Fe_2_O_4_ material disappeared, which may be attributable to its relatively small ratio and its presence in the internal core of the composite matrix. Similar findings were obtained and reported in our previous studies.^33–36^ For the CDs-amorphous sample, a single characteristic broad peak was recorded at nearly 2*θ* = 21.80° (002), indicating the formation of amorphous carbon material.^37^ For the CDs-nanocomposite, same peaks of bare nanocomposite were recorded, because the single broad peak of CDs was suppressed due to its amorphous nature.

#### 3.1.2 SEM, EDX analysis, and elemental mapping techniques

**Fig. 3:**
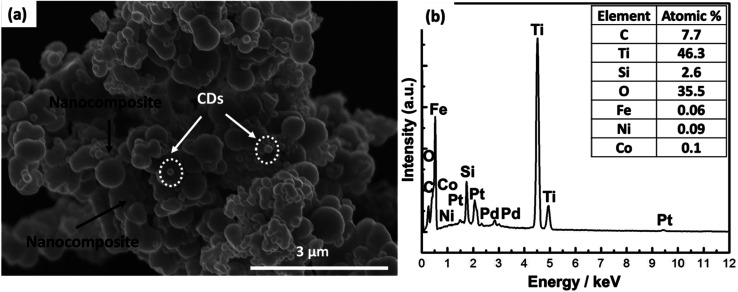
(a) SEM analysis of CDs-nanocomposite and (b) EDX atomic analysis.

SEM, EDX analysis, and elemental mapping were performed to analyze the surface morphology, atomic composition, purity, and homogenous distribution of the elements forming the nanocomposite, as shown in Fig. 3. Analysis of the external morphology of the prepared sample confirmed the homogenous distribution of CDs over the surface of the composite matrix, as exhibited in Fig. 3(a). Elemental mapping techniques showed all the elements (C, Ti, Si, O, Fe, Ni, and Co) forming the nanocomposite in a homogenous distribution, as illustrated in Fig. S1.† Finally, EDX analysis confirmed the purity of the prepared nanocomposite by revealing a lack of foreign elements. In addition, the atomic analysis revealed that Ti and O (forming the TiO_2_ layer) possessed the highest atomic ratios, and the internal Co, Ni, and Fe elements (forming Co_0.5_Ni_0.5_Fe_2_O_4_) possessed the lowest atomic ratios, as presented in Fig. 3(b). This result is in good agreement with our XRD analysis.

#### 3.1.3 BET surface area and BJH pore size distribution analysis

**Fig. 4:**
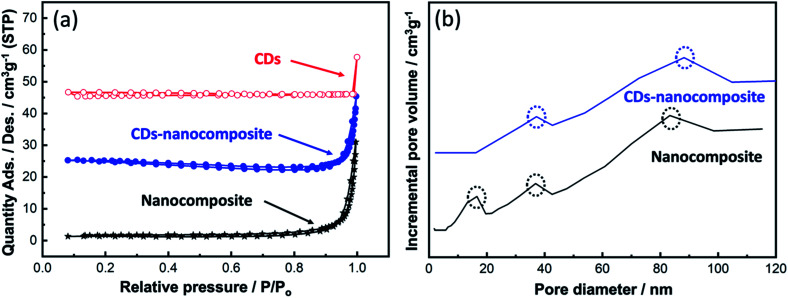
(a) BET surface area analysis of CDs, nanocomposite, and CDs-nanocomposite, and (b) BJH pore size distribution analysis.

The BET surface area and BJH pore size distribution of the synthesized samples were investigated as shown in Fig. 4(a) and (b). According to the IUPAC classification, all samples possessed type (III) adsorption isotherms, which indicate weak interactions between the adsorbate and the prepared adsorbent, as exhibited in Fig. 4(a). In this case, adsorption occurred when the N_2_ interaction with an adsorbed layer was greater than its interaction with the surface of the synthesized samples.

At higher relative pressures (0.87–1), sharp capillary condensation occurred, corresponding to the formation of macropores. The BET surface area, pore volume, and pore area values are summarized in Table 1. Upon CDs loading onto the composite matrix, the BET surface area declined which can be the result of increasing the particle size of the new CDs-nanocomposite. While Fig. 4(b) illustrates the BJH pore size distribution. All samples exhibited a broad, multimodal pore size distribution, confirming the existence of two types of pores: mesopores (2–50 nm) and macropores (>50 nm).

**Table 1:** BET surface area, pore volume, and pore area of CDs, nanocomposite, and CDs-nanocomposite

No	Sample	BET surface area (m^2^ g^−1^)	Pore volume (cm^3^ g^−1^)	Pore area (m^2^ g^−1^)
1	CDs	4.2 ± 2.5	< 0.001	< 0.001
2	Nanocomposite	3.3 ± 0.2	0.001	3.5
3	CDs-nanocomposite	1.2 ± 0.2	0.004	7.8

#### 3.1.4 TEM, HR-TEM, and selected area electron diffraction (SAED) analysis

**Fig. 5:**
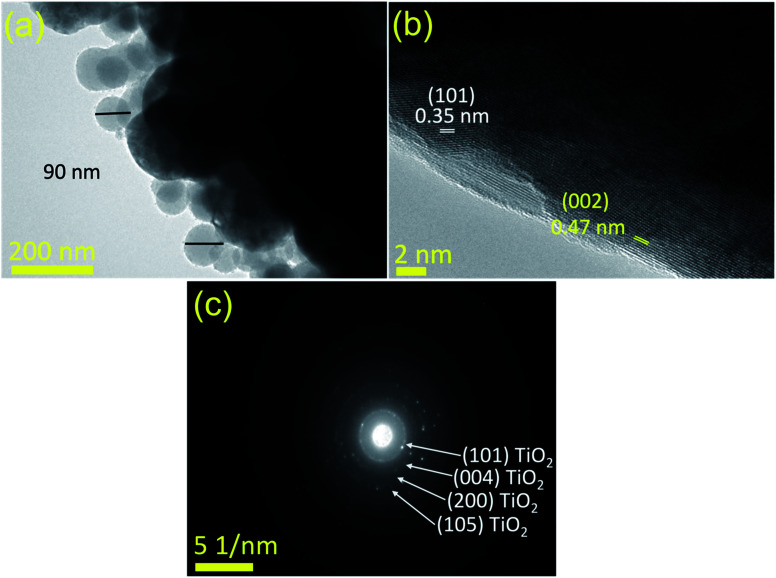
(a) TEM analysis of CDs-nanocomposite, (b) HR-TEM analysis, and (c) SAED pattern.

TEM, HR-TEM, and SAED analyses were performed to calculate the average diameter of the prepared composite matrix, the d-spacings of the lattice planes, and to confirm the formed phase and crystallinity of the synthesized samples, as shown in Fig. 5(a)–(c). Fig. 5(a) displays a TEM micrograph of the CDs-nanocomposite where CDs are distributed uniformly over the external surface of the composite matrix. This is similar to the observation made using SEM. Before CD loading, the average diameter of the matrix was approximately 90 ± 11 nm, and it possessed a spherical shape. After CD loading, the diameter increased. At higher relative pressures, condensation took place in the interparticle voids, which is consistent with the BET results in Fig. 4(a).

Fig. 5(b) reveals the lattice fringes in HR-TEM images, which confirms the conjugation between the predominant anatase TiO_2_ nanoparticles of the matrix and the loaded CDs, characterized by the d-spacing of (002) planes at 0.47 nm. The SAED pattern in Fig. 5(c) shows only the fringes of anatase TiO_2_ and is consistent with the XRD analysis in Fig. 2.

#### 3.1.5 Raman analysis

**Fig. 6:**
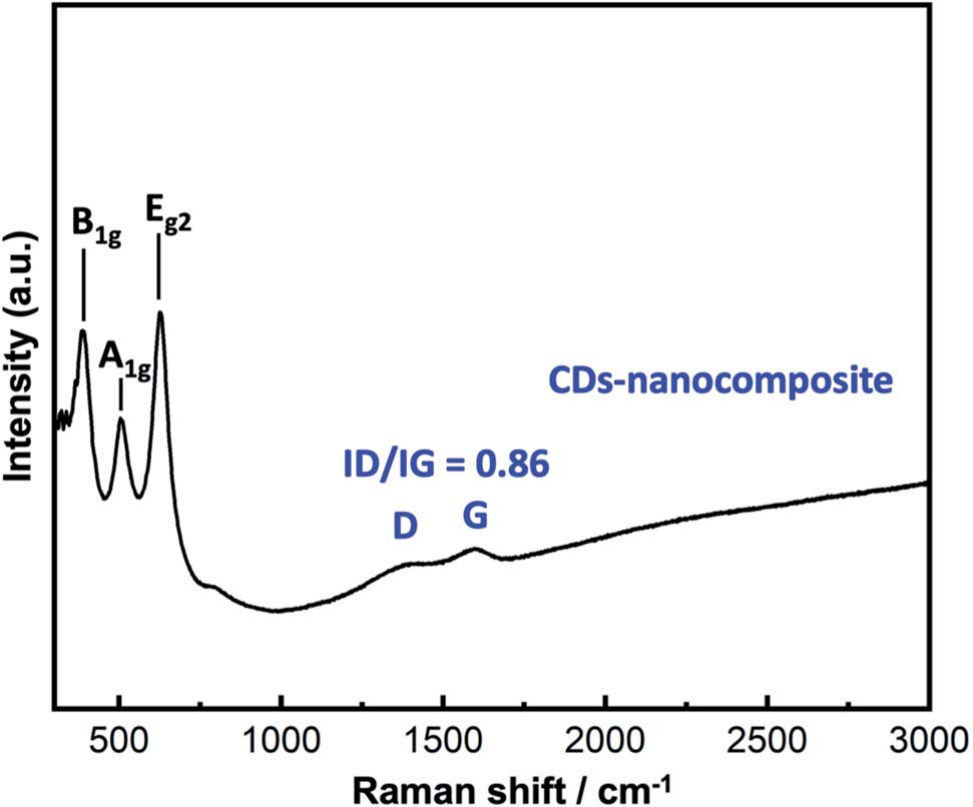
Raman spectra of the nanocomposite and CDs-nanocomposite.

The chemical structure and molecular interactions of the nanocomposite and CDs-nanocomposite were analyzed *via* Raman analysis. Fig. 6 shows the results. As anatase TiO_2_ is the main component of the nanocomposite, three Raman-active bands linked to anatase TiO_2_ are detected at 293.4, 515.7, and 638.6 cm^−1^ corresponding to B_1g_, A_1g_, and E_2g_ modes, respectively. Upon loading CDs into the composite matrix, the characteristic D and G bands attributed to carbon materials appeared at approximately 1379.6 and 1589.3 cm^−1^, respectively. The D band represents sp^3^ defect sites due to grain boundaries and vacancies, while the G band corresponds to the scattering of E_2g_ phonons from sp^2^ C atoms. The *I*_D_/*I*_G_ value was approximately 0.8666.

### 3.2. Electrochemical measurements

**Fig. 7:**
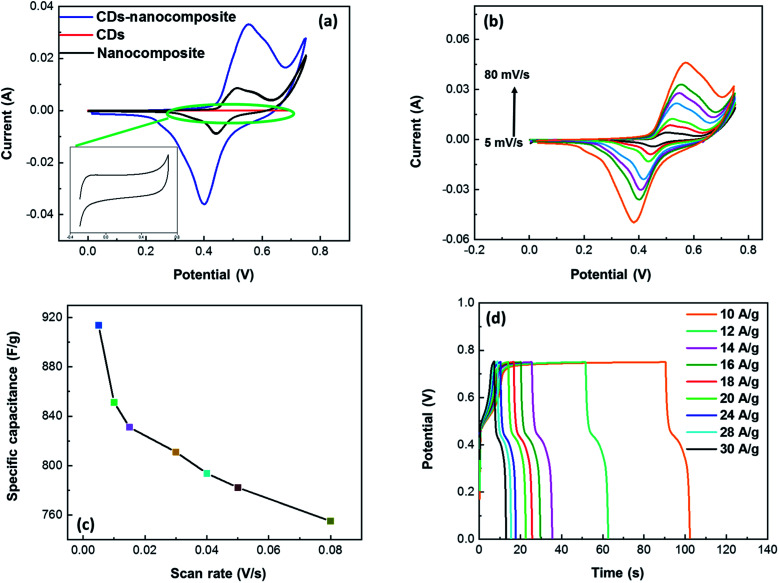
The electrochemical performance of CDs-nanocomposite on NF electrode material (a) comparison of the CV curve of CDs, nanocomposite (inset), and CDs-nanocomposite at a scan rate of 50 mV s^−1^ (b) CV curves of CDs-nanocomposite at various scan rates (c) specific capacitance of CDs-nanocomposite at various scan rates and (d) galvanostatic charge–discharge curves of CDs-nanocomposite at various current densities.

Fig. 7(a) shows a comparison between the CV curves of the CDs, nanocomposite, and CDs-nanocomposite at a scan rate of 50 mV s^−1^ within a potential window of 0–0.75 V. It is accepted that the area of the CV curve is directly proportional to the specific capacitance value. Fig. 7(a) shows the area and current of the CV curve of the CDs-nanocomposite are much greater than that of the CDs and nanocomposite CV curves, which emphasizes the enhanced specific capacitance of the CDs-nanocomposite. Moreover, the CDs curve displays a rectangular shape that reflects EDLC behavior. The shape of the CDs-nanocomposite curve illustrates two redox peaks due to faradaic processes as shown in the following equation (eqn (1)),^38^ which confirms the presence of the composite and its PC mechanisms.^39,40^

1 TiO_2_^−^M^+^ ↔TiO_2_ + M^+^ + e^−^

where M^+^ indicates the protons and alkali metal cations (Na^+^, Li^+^, and K^+^) in the electrolyte.

Fig. 7(b) further demonstrates the properties of the electrode material, illustrating the CV curves of the CDs-nanocomposite tested at different scan rates ranging from 5 to 80 mV s^−1^. All the curves possess two redox peaks. As the scan rate increases, the oxidation peak of the CDs-nanocomposite shifts to a higher potential, and the reduction peak shifts to a lower potential. This can be ascribed to the relatively slow kinetics of the electrochemical reactions at higher scan rates and suggests that the electron transfer process is quasi-reversible or reversible.^41,42^ In addition, as the scan rate increases, the height of the redox peak also increases, and, consequently, the area of the CV curve increases, indicating good electron conduction.^43^ There is no substantial change in the shape of the curves even at the highest scan rate (80 mV s^−1^), which suggests that the CDs-nanocomposite has fast charge transfer properties.^44^Fig. 7(c) shows the specific capacitance of the CDs-nanocomposite at different scan rates. These values were calculated using the following equation:^45^2
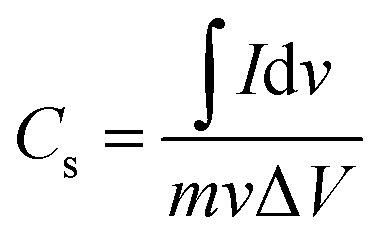
where *I* is the current, *v* is the potential, d*ν* is the potential window, *ν* is the scan rate, and *m* is the mass of the electroactive materials on the electrode. The specific capacitance values were found to be 913.7, 851.3, 831.1, 811.0, 793.7, 782.2, and 755 F g^−1^ at scan rates 5, 10, 15, 30, 40, 50, and 80 mV s^−1^, respectively.

The curves at different current densities ranging from 10 to 30 A g^−1^ were compared, as illustrated in Fig. 7(d), by controlling the measured currents of the CDs-nanocomposite according to the mass of active substance coated on the NF substrate. These values were calculated using the following equation:^46^3
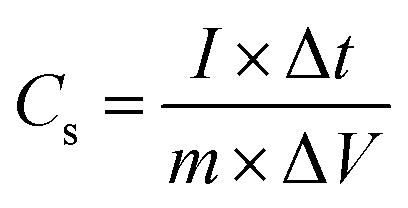
where *C*_s_ is the specific capacity, *m* is the mass of active substance coated on the substrate, *I* is the discharge current, and Δ*t* is the discharge time.^47^ The specific capacitance values of the CDs-nanocomposite were found to be 837, 741.8, 710.7, 654.2, 594.3, 561.5, 515, 483.2, and 462.6 F g^−1^ at current densities 10, 12, 14, 16, 18, 20, 24, 28, and 30 A g^−1^, respectively.

**Fig. 8:**
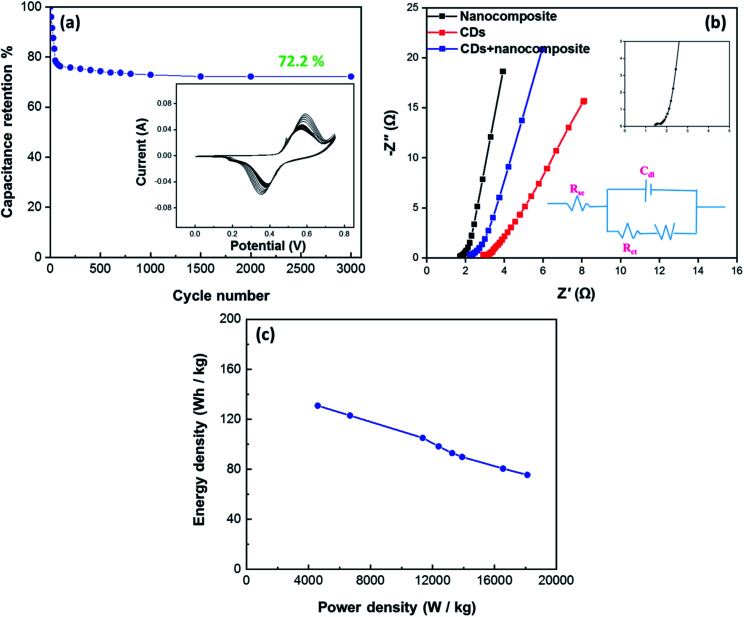
(a) Long-life cyclic stability (over 3000 cycles) of CDs-nanocomposite at a scan rate of 80 mV s^−1^ in 1 M KOH electrolyte (inset is the CV curve for the initial and final few cycles of CDs-nanocomposite), (b) Nyquist plot of nanocomposite, CDs-nanocomposite and CDs on NF (inset figure is the high frequency region of CDs-nanocomposite at the top, and EIS equivalent circuit at the bottom) and (c) Ragone plots of the CDs-nanocomposite.

The cycling stability of the electrode material was analyzed using the CV technique continuously for 3000 cycles at a constant scan rate of 80 mV s^−1^ within a potential window of 0–0.75 V to evaluate the electrochemical behavior of the CDs-nanocomposite further. The results presented in Fig. 8(a) show that the CDs-nanocomposite electrode material delivers 72.2% capacitance after 3000 cycles, signifying its remarkable long-life cyclic stability.

More helpful technique to understand the electrochemical performance and the energy storage mechanism for CDs-nanocomposite as hybrid SCs is electrochemical impedance spectroscopy (EIS) technique. EIS is a very significant technique to analyze the electrochemical behavior occurring at different frequencies in SCs. The EIS provides a total overview of all the frequency behavior of a system and gives information about the capacitive behavior, the resistance, and the diffusion-limited processes. EIS techniques make it easy to differentiate between EDLC and faradaic reaction capacitance in redox-active materials since these two processes occur at different frequencies. The ideal Nyquist plot produced by EIS consists of a semicircle at high frequency, a tilted linear variation of the impedance in the middle frequency range, and a vertical tail at low frequency.^48,49^

Fig. 8(b) shows the Nyquist plots for the CDs, nanocomposite, and CDs-nanocomposite obtained in the frequency range of 1.0 to 200 kHz with an ac voltage amplitude of 10 mV. Semicircles are as the charge transfer resistance (*R*_ct_) is the charge transfer resistance at the electrode–electrolyte interface, and *R*_s_ is the solution resistance, calculated from the semicircle's intercept with the *x*-axis.^50,51^ It can be noticed that, *R*_s_ of CDs-nanocomposite gives intermediate value of 1.5 Ω compared to nanocomposite and CDs. This value of *R*_s_ indicates the difference in conductivity of the electrode materials, the lower *R*_s_ value of CDs-nanocomposite shows the higher conductivity due to a good contact between composite with highly conductive CDs and also short electron path-length as the presence of CDs helps the electrons to migrate to the surface of the electrode active materials which is favorable to redox reactions.^41^ The very small semicircle that appears in the region of high frequency has very low diameter, showing low *R*_ct_ (0.4 Ω). The line with a sharp slope at low frequency is specific for the electrode capacity, that looks vertically in ideal capacitors.^52^ As shown in Fig. 8(b), the linear part of the CDs-nanocomposite curve looks more vertical, with a smaller practical impedance value, and its shape looks closer to the ideal form. Therefore, it can be noted that the modification of the nanocomposite with CDs enhances the electrochemical performance of hybrid SCs.

Fig. 8(c) shows the Ragone plot (power density *vs.* energy density) of the CDs-nanocomposite. The values of energy density and power density were calculated from the following equations:^53,54^4
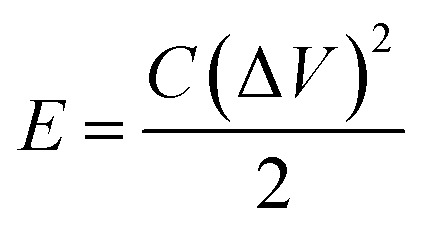
5
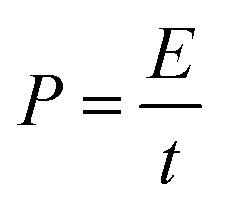
where *E* (W h kg^−1^) is energy density of the supercapacitor, *C* (F g^−1^) is the total specific capacitance, *P* (kW kg^−1^) is power density, and *t* is the discharge time (h). Table 2 details the energy density and specific capacitance of the TiO_2_–carbon-based material synthesized in this work compared with those synthesized in other studies.

**Table 2:** Comparison of the electrochemical performance (specific capacitance and energy density) of TiO_2_–carbon-based materials synthesized by different methods for SC application

Electrode material	Method	Electrolyte	Specific capacitance		Power density	Energy density	Ref
TiO_2_–CNT film	Electrochemical deposition	PVA–H_2_SO_4_		345.7 F g^−1^ at 1 A g^−1^	9.4 kW kg^−1^	82.5 W h kg^−1^	55
CNT/TiNiW	Dr Blade technique	PVA/H_3_PO_4_		549.1 F g^−1^ at 1 A g^−1^	—	336.7 W h kg^−1^	56
Co_3_O_4_/TiO_2_/activated carbon on NF	Simple solgel	6 M KOH		946 F g^−1^ at 5 mV s^−1^	—	—	57
SWCNTs/TiO_2_	Hydrothermal	PVA/H_2_SO_4_		144 F g^−1^ at 1 A g^−1^	62.5 W kg^−1^	20 W h kg^−1^	58
CDs/TiO_2_	Chemical reduction & pyrolysis	2 M KOH		2200 mF cm^−2^ at 5 mV s^−1^	635 µW cm^−2^	51.3 µW h cm^−2^	21
CDs/PPy/TiO_2_ nanotube arrays	Anodization	0.15 M pyrrole monomer & 0.1 M (LiClO_4_)		482 F g^−1^ at 0.5 A g^−1^	150 W kg^−1^	42.5 W h kg^−1^	59
CDs-nanocomposite	Layer-by-layer and hydrothermal methods	1 M KOH		913.7 F g^−1^ at 5 mV s^−1^	20 kW kg^−1^	130.7 W h kg^−1^	This work

## 4 Conclusion

To summarize, in this study, a TiO_2_-based nanocomposite (Co_*x*_Ni_1−*x*_Fe_2_O_4_, *x* = 0.5/SiO_2_/TiO_2_) was synthesized using a simple layer-by-layer approach. It was then enhanced with CDs synthesized using a one-pot hydrothermal method, loaded on NF substrate, and investigated as a potential electrode material for hybrid SCs. The structure and morphology of the CDs-nanocomposite were analyzed using various techniques, such as XRD, BET, Raman spectroscopy, SEM, EDS, and TEM. The calculated specific capacitance value of the CDs-nanocomposite was 913.7 F g^−1^ at a scan rate of 5 mV s^−1^, and it demonstrated good cyclic stability over 3000 cycles with capacitance retention of 72% and a superior energy density value of 130.7 W h kg^−1^. In conclusion, we suggest that the CDs-nanocomposite developed in this study has the potential to enhance the electrochemical performance of hybrid SCs and that the simple synthesis method can be applied to similar TiO_2_ nanocomposites to create favorable electrode materials for advanced energy storage systems.

## Conflicts of interest

There are no conflicts to declare.

## Supplementary Material

RA-011-D1RA08045H-s001
