# Supplementary material for: Carbon dots conjugated nanocomposite for the enhanced electrochemical performance of supercapacitor electrodes
Source: RSC Adv. 2021 Dec 13;11(63):39636–45. doi: 10.1039/d1ra08045h (PMC9044567; doi:10.1039/d1ra08045h)
Supplement: RA-011-D1RA08045H-s001 [file RA-011-D1RA08045H-s001.pdf]

Electronic Supplementary Material (ESI) for RSC Advances.
This journal is © The Royal Society of Chemistry 2021

Carbon dots conjugated nanocomposite for the enhanced electrochemical performance of supercapacitor electrodes

Sally M. Youssry[1]†, M. Abd Elkodous[2]†, Go Kawamura[2], and Atsunori Matsuda[2,*]

[1] Department of Chemistry, Faculty of Science, Tanta University, Tanta, 31527, Egypt.

[2] Department of Electrical and Electronic Information Engineering, Toyohashi University of Technology, 1-1 Hibarigaoka, Tempaku-cho, Toyohashi, Aichi 441-8580, Japan.

† Equal Contribution

***Corresponding Author**

Prof. Dr. Eng. Atsunori Matsuda, Professor, Toyohashi University of Technology, 1-1 Hibarigaoka, Tempaku-cho, Toyohashi, Aichi 441-8580, Japan.

Email: matsuda.atsunori.hh@tut.jp

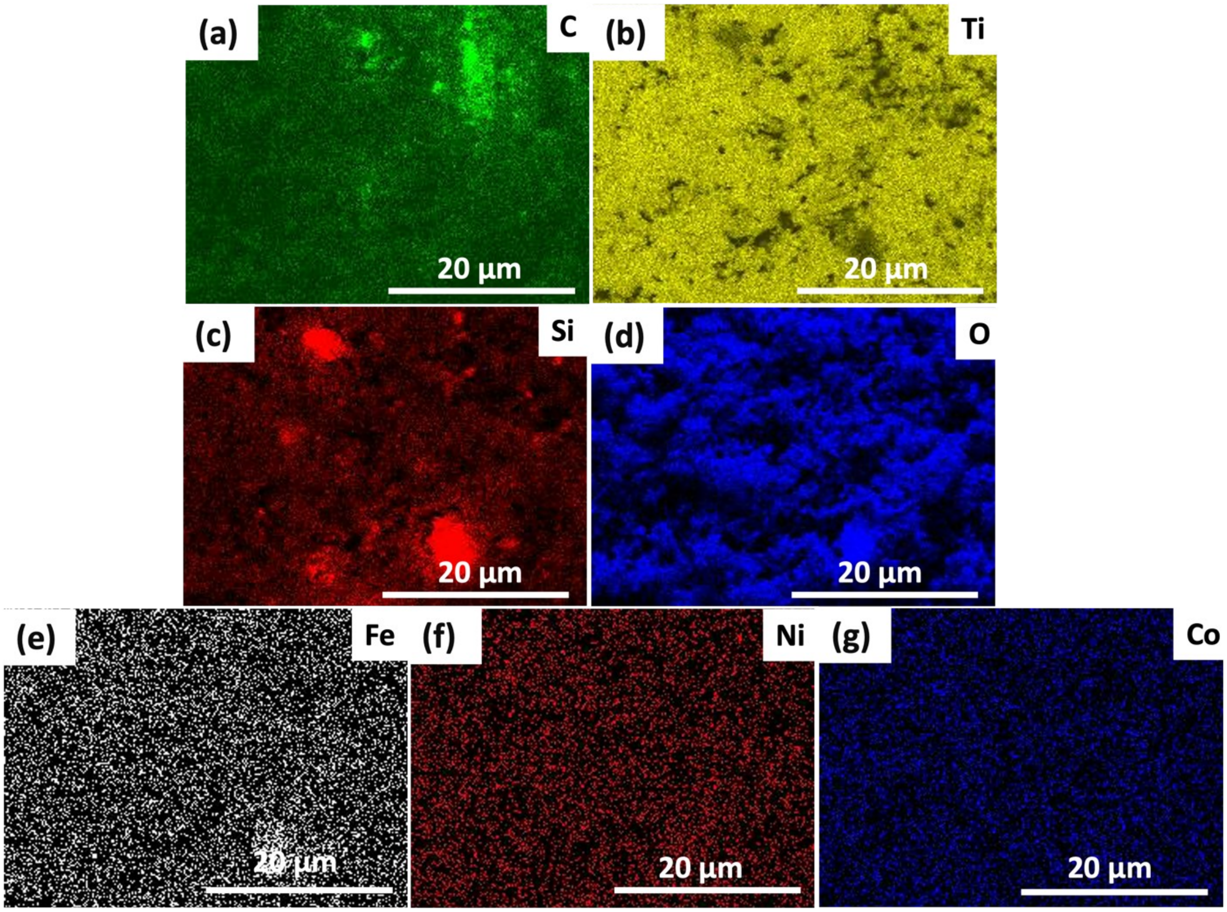

Fig. S.1: Elemental mapping of the prepared CDs-nanocomposite.

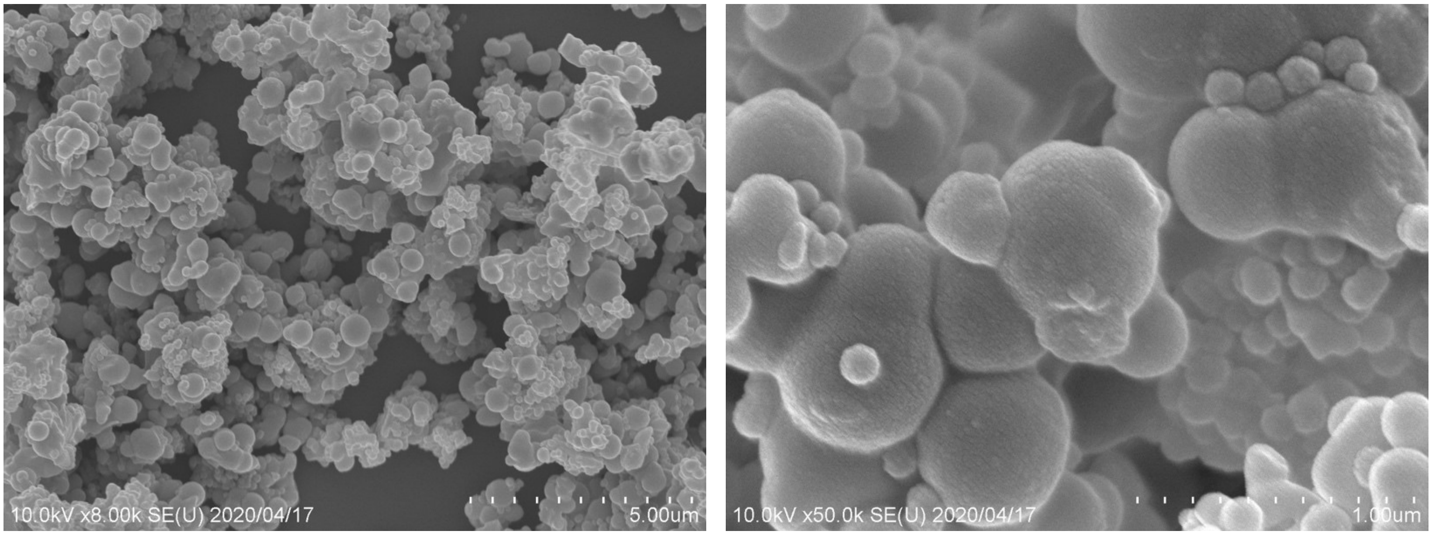

Fig. S. 2: SEM images with different magnifications of the prepared CDs-nanocomposite.

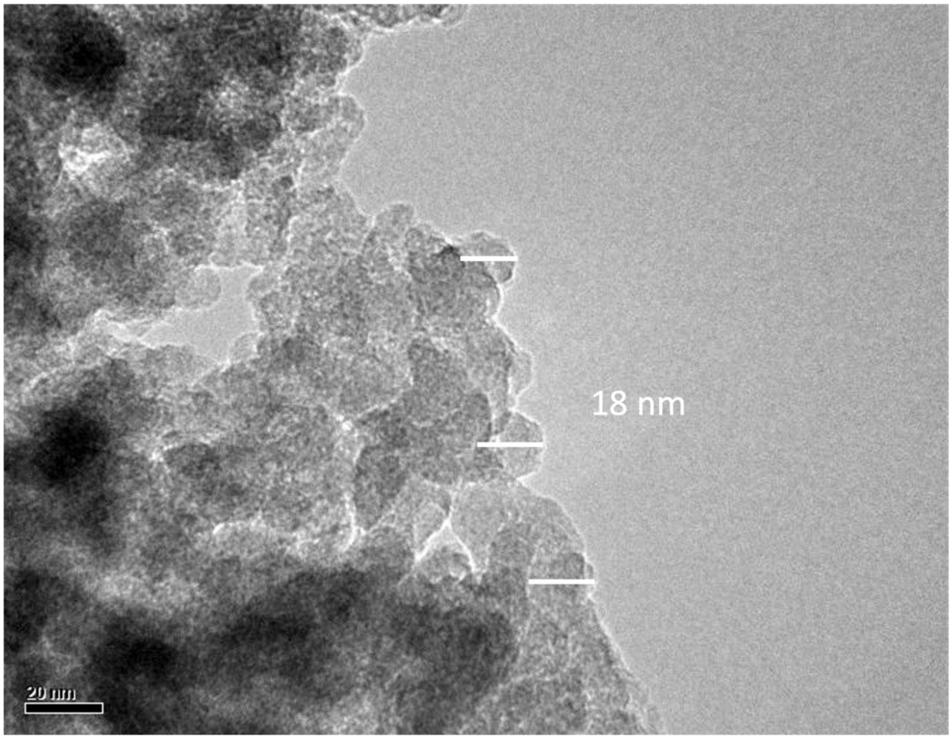

Fig. S. 3: TEM analysis of the prepared CDs showing its average particle size of about 18 nm.
